# 1026. Following the Hoof Prints: Detecting *Coxiella* and *Brucella* infections with A Plasma-based Microbial Cell-Free DNA Next-generation Sequencing Test

**DOI:** 10.1093/ofid/ofab466.1220

**Published:** 2021-12-04

**Authors:** Nicholas R Degner, Ricardo Castillo-Galvan, Jose Alexander, Aparna Arun, Christiaan R de Vries, Ann Macintyre, Bradley Perkins, Asim A Ahmed, Matthew Smollin

**Affiliations:** 1 Karius Inc., San Francisco, California; 2 Karius, Inc., Franklin, Tennessee; 3 Karius, Redwood City, California; 4 Karius, Inc, Redwood City, CA

## Abstract

**Background:**

*Coxiella burnetii* and *Brucella spp*. are zoonotic bacterial pathogens responsible for Q fever and Brucellosis, respectively. Both pathogens have a global distribution and Brucellosis is the most common zoonosis in the world. However, the CDC reports only 80-120 cases of human brucellosis and ~150 cases of acute Q fever annually. The diagnosis of these infections can be limited by: (1) their difficulty to culture; (2) the insensitivity and nonspecificity of serology; (3) the clinical overlap with other infections; and (4) the unreliability of epidemiological exposure history for these zoonoses. Unbiased microbial cell free DNA (mcfDNA) next-generation sequencing (NGS) offers a potential solution to overcome these limitations.

**Methods:**

The Karius Test^TM^ (KT) developed and validated in Karius’s CLIA certified/CAP accredited lab in Redwood City, CA detects mcfDNA in plasma. After mcfDNA is extracted and NGS performed, human reads are removed, and remaining sequences are aligned to a curated database of > 1500 organisms. McfDNA from organisms present above a statistical threshold are reported and quantified in molecules/µL (MPM). KT detections of *Coxiella* and *Brucella* were reviewed from August 2017 - present; clinical information was obtained with test requisition or consultation upon result reporting.

**Results:**

KT detected 8 cases of *Coxiella burnetii* (1735 MPM +/- 3000) and 5 cases of *Brucella melitensis* (avg 296 MPM +/- 223) (Table 1), representing approximately 1-2% of all detections in the US during this period. All of the Coxiella detections were in adults (100% male) with 5 cases of fever of unknown origin, 2 cases of culture-negative endocarditis and one case of endovascular graft infection. *Brucella* detections occurred in 3 adults and 2 children (60% male), 3 with exposure to unpasteurized dairy and included 3 cases of spine infection (2 vertebral osteomyelitis, 1 epidural abscess).

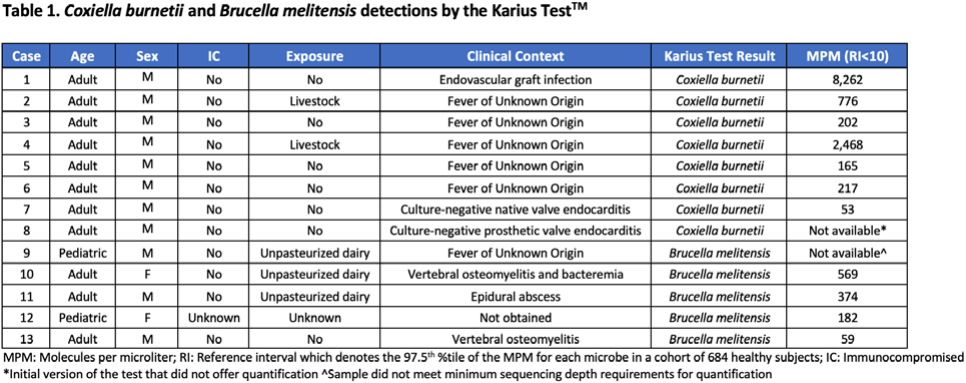

**Conclusion:**

Open-ended, plasma-based mcfDNA NGS provides a rapid, non-invasive test to diagnose diverse clinical manifestations of zoonotic infections such as Q fever and Brucellosis against competing broad differential diagnoses. Furthermore, these cases highlight the potential of the KT to diagnose infections caused by fastidious/unculturable pathogens with cryptic clinical presentations.

**Disclosures:**

**Nicholas R. Degner, MD, MPH, MS**, **Karius Inc.** (Employee, Shareholder) **Ricardo Castillo-Galvan, MD MPH**, **Karius Inc.** (Consultant) **Jose Alexander, MD, D(ABMM), FCCM, CIC, SM, MB(ASCP), BCMAS**, **Karius** (Employee) **Aparna Arun, MD**, **Karius** (Employee) **Ann Macintyre, DO**, **Karius, Inc.** (Employee) **Bradley Perkins, MD**, **Karius, Inc.** (Employee) **Asim A. Ahmed, MD**, **Karius, Inc.** (Employee) **Matthew Smollin, PharmD**, **Karius, Inc.** (Employee)

